# The Story of the Insane from Year to Year

**Published:** 1901-10-12

**Authors:** 


### THE STORY OF THE INSANE FROM YEAR TO YEAR.

(Thirteenth Series.)

### DERBY COUNTY ASYLUM.

This asylum contained 591 patients on January 1st, 1899, and 597 on December 31st. There were 156 first admissions 27 readmissions, 66 recoveries, 17 discharged relieved, 7 discharged not improved, and 81 deaths. The average daily number resident was 588, and the total number under care was 770. The recoveries stand at 35·8 per cent. of the admissions, and the deaths at 13·7 per cent. of the average number resident. Of the year's deaths 34 were caused by cerebral and spinal diseases, 24 by consumption, 1 by pneumonia, and 10 by colitis and enteritis. Of the year's admissions the insanity was caused by moral causes in 41 cases, by intemperance in drink in 27, and by hereditary influence in 27. In 26 cases no cause is given. Dr. Legge refers to the high death-rate from consumption, and says "it is very disappointing when we consider the many sanitary improvements—new drainage system, new floors, &c.—which have been carried out; and it was hoped that the number of pulmonary diseases would decline after the introduction of mechanical heating and ventilation." We greatly doubt whether mechanical heating will ever reduce the death-rate from phthisis. Is it not more likely to increase it unless very carefully managed and as an adjunct only? Dr. Legge's remarks upon the colitis epidemic are particularly interesting. The following is a summary of his facts and conclusions:—1. There were 92 cases in about 18 months, with 39 deaths. 2. All the patients, except perhaps 2, acquired the disease in the asylum. 3. Men and women were equally attacked. 4. That the disease may be more common than is supposed, slight cases escaping observation; but the officials were taught to look for the disease, and the 92 cases were all that could be identified. 5. Infection enters by the mouth, and is due to a microbe. 6. Patients on ordinary diet and on sterilised milk are attacked indiscriminately. 7. It rarely, if ever, attacks the staff, but chiefly demented and physically weakened patients. 8. No explanation why it is present at one time and absent at another. 9. No reason to suspect the drainage. 10. Mechanical ventilation of wards has no influence, and the disease appeared equally in the old wards and in the new ones. 11. That the *Bacillus Enteritidis Sporogenes* was found in the intestines in every case, but also in healthy excreta, and abundantly in the ward sweepings. 12. The microbes may be of common or almost universal occurrence, like that of tuberculosis, but may not be sufficiently active to produce the disease except in feeble patients. Dr. Legge sums up by saying "that so far as the cause of the disease is concerned, I have to admit that up to the present I have learned little." We hardly think that Dr. Legge's surmise expressed in No. 12 can be correct, because there are always plenty of demented, dirty, enfeebled cases in all our asylums. We are satisfied that he is right about the drainage and about the diet. Bad drainage does not seem to cause, nor good drainage to prevent the disease, and as regards the diet, experiments have been made in many asylums which seem to demonstrate that no cause has been found therein. We wish that Dr. Legge had seen fit to mention what treatment he found most useful, although, as a rule, we do not approve of medical details in these reports. We are glad to note that attendant William Hill has been granted a pension of £36 a year, and that attendant Henry Wasley has been granted a similar sum. The committee report that the management of the asylum has been quite satisfactory. The weekly rate of maintenance was 10s.

### HOLLOWAY SANATORIUM FOR THE INSANE AT VIRGINIA WATER.

On January 1st, 1899, this hospital for the insane contained 370 patients, and on December 31st the numbers had risen to 381. There were 137 first admissions, 25 readmissions, 61 discharged recovered, 38 relieved, 24 not improved, and 28 died. The average number resident during the year was 377, and a total of 520 were under treatment. Excluding transfers, the percentage of recoveries was 49·19 of the admissions, and the percentage of deaths was 7·43 of the average number resident. Of the deaths 16 were caused by cerebral and spinal diseases; 4 by consumption, and 2 by decay of old age. Moral causes, either as predisposing or as exciting, are stated to have caused the insanity in 54 instances; intemperance in drink in 24, hereditary influence in 10, and in 31 the cause of insanity was unknown. There were 49 voluntary boarders admitted during the year, which is a convincing proof of the estimation in which the hospital is held by the people who know it. At the beginning of the year Dr. Rees Philipps, who had been medical superintendent of the asylum from its opening, resigned, owing to ill-health, and was granted a liberal pension. The committee say that much of the success of the hospital was due to Dr. Philipps' energy and management; and Dr. Moore, who was appointed successor, pays a graceful tribute to his former colleague, who, he says, had taken constant and personal interest in the welfare of the patients and staff, and that "there was universal regret when he left."

### DUNDEE ROYAL ASYLUM.

This is one of the asylums whose statistics follow an arbitrary year from June 19th of one year to June 18th of the next year; and it would be very desirable to adopt the usual method from January 1st to December 31st. No good end is served, but some confusion caused, by the other course. On June 19th, 1899, there were in the asylum 418 patients, and on June 18th, 1900, the numbers had fallen to 402. There were 83 first admissions, 22 readmissions, 49 discharged recovered, 30 discharged relieved, 4 not improved, and 41 died. The recoveries stand at 43·81 per cent. of the admissions, and the deaths at 9·97 per cent. of the average number resident—the former percentage being above the public asylum average, and the latter just about the average. Of the deaths 18 were caused by cerebral and spinal diseases, and 4 by consumption. Various moral causes are put down as being the predisposing cause of insanity in 11 of the year's admissions, and as the exciting cause in 17. Hereditary tendency was the assigned cause in 14 instances, and drink in 18. There were 21 private patients among the 105 admissions. A building for the treatment of this class of patients is being erected, and on the date of the report was rapidly approaching completion. The training of nurses and attendants is continued, and four candidates obtained the nursing certificate of the Medico-Psychological Association, making a total of 36 of Dr. Rorie's staff who are in possession of it.

### CARLOW DISTRICT ASYLUM.

The limits of accommodation are given at 342 beds, but 346 patients were in residence at the beginning of 1899, and at the end of the year the numbers had risen to 368. There were 66 first admissions, 19 readmissions, 33 discharged recovered, 7 discharged relieved, 4 not improved, and 19 died. Of the year's deaths 11 were caused by cerebral and spinal diseases, 4 by consumption, and 1 by pneumonia. The recoveries, calculated on the admissions, stand at 38·8 per cent., and the deaths, calculated on the average number resident, at 5·4 per cent. Table XVII. shows that in 9 of the admissions various moral causes were assigned as causing insanity, in 8 drink, and in 32 hereditary influence. Dr. O'Meara has increased the amount of liberty given to the patients and no escape occurred during the year, nor was there any suicide, and the only accident chronicled is that of a fractured clavicle. Restraint was used in three cases and seclusion twice. The committee do not publish a report of the asylum. Dr. Courtenay, the Inspector of Lunatics, says that the "additional accommodation has not been given over for the use of the patients," and that "it is hopeless to predict anything of the future as regards the completion of the works here," from which we gather that things in Carlow do not move very fast. The annual cost per head was £24 16s. 11d.

### WEST RIDING ASYLUM AT WAKEFIELD.

There were 1,423 patients in this asylum on January 1st, 1899, and the number had risen by 4 on December 31st. The first admissions numbered 245, the readmissions 47, the recoveries 119, the discharged relieved 44, and the deaths 123. The total number of persons under care during the year was 1,707. Calculated on the admissions the recoveries stand at 40·75 per cent., and calculated on the average number resident the deaths stand at 8·65 per cent., the former being a little above the average and the latter a little below it. Of the year's deaths we notice that 49 were caused by cerebral and spinal diseases, 19 by consumption, 8 by pneumonia, 2 by fibroid phthisis, 3 by bronchitis, pulmonary œdema, and pleuro-pneumonia, and 2 by general tuberculosis. Here we have 23 deaths during the year caused by tubercular disease, and Dr. Lewis gives us no explanation, but it is shown that the general mortality was lower in 1899 than it had been in any year since 1846. Turning to Table X. we learn that various moral causes played some part in the causation of the insanity in 61 of the year's admissions, that intemperance in drink was the cause in 75, hereditary tendency in 94, previous attacks in 53, and, so carefully has this table been prepared, that in 5 only was the cause unknown. For a long time the Berry Wood Asylum was the only county asylum in England which treated idiot children apart from the adults. Lately two other counties have made provision for these cases, and now the West Riding is following the good example by the purchase and fitting up of Stanley Hall—a building close to the Wakefield Asylum. The accommodation will be for "over 60 inmates," but as idiots are to be received from the three West Riding Asylums, it is evident that the accommodation is ridiculously insufficient. Nothing short of 200 beds should be thought of and it is equally certain that no such number could be safely added to any asylum already containing nearly 1,500 patients; the correct solution would therefore have been an entirely separate establishment. The epileptics are also to be separated from the other patients at the Wakefield Asylum; and there is much to be said in favour of this idea provided it be remembered that epileptics themselves need much classification. Dr. Lewis speaks highly in favour of the epileptic colony system, which has certainly met with considerable success; and yet the asylum physician with many cases studding his memory will feel inclined to doubt whether any epileptic who has once been insane is ever again to be trusted. Nonconformist services for 200 patients are held every Saturday in one of the day-rooms, and we are told that "much satisfaction has been expressed with this new departure." It is not easy to see why such a number of patients should not have the use of the chapel on a Sunday when not wanted for the other services. The committee say that "the management of the institution is highly creditable to Dr. Bevan Lewis." The weekly charge to the Unions is 9s. 4d. per head.

